# Fanconi anemia gene variants in therapy-related myeloid neoplasms

**DOI:** 10.1038/bcj.2015.44

**Published:** 2015-07-03

**Authors:** M T Voso, E Fabiani, Z Zang, L Fianchi, G Falconi, A Padella, M Martini, S Li Zhang, R Santangelo, L M Larocca, M Criscuolo, A La Brocca, I Cutcutache, S Rozen, G Simonetti, M Manfrini, G Martinelli, S Hohaus, G Leone, P Tan, D G Tenen

**Affiliations:** 1 Department of Hematology, Università Cattolica Sacro Cuore, Rome, Italy; 2 Department of Biomedicine and Prevention, Tor Vergata University, Rome, Italy; 3 Department of Cellular and Molecular Research, National Cancer Centre, Singapore, Singapore; 4 Department of Haematology/Oncology, L and A Seragnoli, Universita' di Bologna, Bologna, Italy; 5 Department of Pathology, Università Cattolica Sacro Cuore, Rome, Italy; 6 Cancer and Stem Cell Biology, Duke-NUS Graduate Medical School, Singapore, Singapore; 7 Department of Microbiology, Universit' Cattolica Sacro Cuore, Rome, Italy

Therapy-related myeloid neoplasms (t-MN) include myelodysplastic syndromes (MDS) and acute myeloid leukemias (AML) occurring as a late effect of chemotherapy and/or radiotherapy for a primary malignancy or for autoimmune diseases.^1, 2^ The incidence of this complication has been raising in the past years because of the prolonged survival and the higher number of treated patients. Still, <5% of patients exposed to cytotoxic drugs and radiotherapy develop a t-MN, suggesting an underlying individual susceptibility. Primary malignancies most frequently associated to t-MN are breast cancer and lymphoproliferative diseases. Other recurrent clinical characteristics are presence of multiple primary neoplasms in the same individual and cancer familiarity.^2^ So far, higher frequency of single-nucleotide variants of detoxification and DNA-repair enzymes, alone or in association, have been reported in t-MN, but none has been validated as significant risk factor in large patient groups.^3, 4, 5, 6^

The association of breast and other cancers to myeloid neoplasms is frequent in Fanconi anemia (FA), a childhood syndrome characterized by chromosomal instability, developmental abnormalities, aplastic anemia and by predisposition to cancer, particularly gynecological, head-neck and gastrointestinal.^7, 8, 9^ FA is associated to the occurrence of bi-allelic loss-of-function mutations in the family of FANC genes, comprising 16 DNA-repair genes. Mutations in FANCA, FANCC and FANCG are the most common, and account for ~85% of the FA patients. Less than 5% of FA patients do not appear to have mutations in these 16 genes, indicating that additional genes may be involved. Carriers of germline homozygous mutations of at least five of the sixteen FA genes, including *FANCD1/BRCA2*, *FANCJ/BRIP1*, *FANCN/PALB2*, *FANCO/RAD51C* and *FANCQ/ERCC4*, are at a higher-risk to develop cancer.

So far, mutations in FA genes have been rarely described in hematological malignancies outside the syndromic picture of FA.^10, 11, 12^ We were interested in the prevalence of FA variants in t-MN following cytotoxic treatment for breast cancer and lymphoproliferative diseases, compared with *de novo* acute myeloid leukemia (dnAML).

We studied 37 patients with a t-MN, diagnosed at our Department of Hematology between November 1995 and January 2012. Table 1 shows patient characteristics. Median age was 63 years (range 30–78). According to the proportion of blasts, there were 19 t-MDS and 18 t-AML. Cytogenetic analysis of bone marrow mononuclear cells obtained at t-MN diagnosis revealed clonal aberrations in 24 out of 32 available samples (75%). The primary malignancy was Hodgkin lymphoma in 7 patients, non-Hodgkin lymphoma in 12 patients and breast cancer in 18 patients (associated with a second malignancy in five patients). As a control, 24 dnAML patients, of a median age of 61 years (range 40–78 years), were studied at the Istituto Seragnoli of the University of Bologna. Patients gave informed consent to the study and the protocol received approval from the local Ethical Committees.

Bone marrow mononuclear cells were separated at the time of initial t-MN diagnosis using Ficoll Gradient centrifugation (Cedarlane, ON, Canada), and DNA was extracted by using the QIAamp DNA Mini Kit (Qiagen AG, Hilden, Germany). DNA extracted from different tissues of the same individuals served as germline controls and included: lymphnode biopsies or breast specimens (neoplastic and non-neoplastic) collected at the time of the primary cancer diagnosis, buccal brush cytology or hair follicles collected at the time of t-MN or dnAML diagnosis, peripheral blood or bone marrow samples collected during follow-up of the primary malignancy or at the time of complete remission.

We selected 14 Fanconi pathway genes (*FANCD1* (*BRCA2*), *FANCJ* (*BRIP1*, *BACH1*), *FANC1*, *FANCA*, *FANCB*, *FANCC*, *FANCD2*, *FANCE*, *FANCF*, *FANCG* (*XRCC9*), *FANCL* (*PHF9*), *FANCM*, *FANCN* (*PALB2*) and *RAD51C*). For t-MN, targeted gene enrichment was performed using the Agilent HaloPlex system (Agilent Technologies Inc, Santa Clara, CA, USA). Briefly, following digestion of genomic DNA samples in eight different restriction reactions, each containing two restriction enzymes, we created a library of gDNA restriction fragments. The collection of gDNA restriction fragments was then hybridized to the HaloPlex probe capture library. During the hybridization process, Illumina (San Diego, CA, USA) sequencing motifs including index sequences were incorporated into the targeted fragments. The circularized target DNA-HaloPlex probe hybrids, containing biotin, captured on streptavidin beads were then ligated using a DNA ligase to close nicks in the circularized HaloPlex probe-target DNA hybrids. The captured target DNA was then eluted with NaOH and amplified by PCR. The amplified target DNA was purified using AMPure XP beads (Beckman Coulter Inc, High Wycombe, UK), validated and quantified by microfluidics analysis using the 2100 Bioanalyzer (Agilent Technologies). Samples have been pooled with different indexes for multiplexed sequencing using Illumina Hiseq platform at the Cancer and Stem Cell Biology, Duke-NUS Graduate Medical School in Singapore. Sequencing data were aligned to hg19 using the Burrows-Wheeler Aligner software. Variants were called using the Genome Analysis Toolkit (GATK, v1.0.4333; Beckman Coulter Inc). dnAML samples were studied using 100 bp paired-end whole-exome-sequencing (HiSeq2000, Illumina). Variants were called with the Genome Analysis Toolkit (GATK).

Variants detected by NGS were confirmed by pyrosequencing or Sanger sequencing, using specifically designed oligonucletides targeting the mutated region (Supplementary Table 1). Reagents and conditions were as recommended by the manufacturers (PyroMark Q96 ID, Diatech Pharmacogenetics, Jesi, Italy). A BLAST search on NCBI, Cosmic and UCSC databases was performed for all identified variants. The Polyphen-2 tool (genetics.bwh.harvard.edu) was consulted to define the putative effect of FA discovered variants, predicting the possible impact of the amino acid substitutions on the structure and function of the proteins. Odds ratios, with 95% confidence interval were also calculated.

FA gene variants were frequent in our t-MN patients, with 6 out of 37 patients (16%) carriers of at least one genomic variant, with similar prevalence in t-MN secondary to lymphoproliferative diseases versus solid tumors (4 out of 19 vs 2 out of 18, respectively). We found seven heterozygous FANC variants, including two FANCA (L6F and S90T), three FANCD2 (T1376A, P256S and M1023V), one FANCJ (I364V) and one FANCC (L36F) (Table 2). There were no differences in the time between primary cytotoxic treatment and t-MN diagnosis in carriers of FA variant versus wild-type patients (median, 72 vs 69 months). Six variants were novel, according to the NCBI and Cosmic databases, whereas the FANCA L6F had been previously described in the 1000 Genome project (www.1000genomes.org) and reported in the UCSC Genome Browser in the transcript variant 2 of FANCA mRNA (uc002fow.1). This same variation has been conflictually reported as SNV in the 5′ UTR of NCBI Reference Assembly (*rs189841793*).

To determine whether FANC genetic variants were somatically acquired in t-MN patients, germline material was screened in five patients for whom control tissues were available. The FANC mutation was germline in all cases (5 confirmed in 37 t-MN patients, 13.5%) (Table 2). As a control, the frequency of FANC variant in t-MN was compared with that of 24 dnAML patients, studied using whole-exome-sequencing. We found that 3 out of 24 patients (12.5%) were carriers of a germline FA variant (1 FANCA, 1 FANCL and 1 FANCJ). This translates into a similar prevalence of FA SNV in t-MN and dnAML. Since SNV in FA genes seem frequent both in *dn*AML and in t-MN, we suggest that these variants may have a role as risk factors in the development of myeloid neoplasms.

As all FANC variants were novel, further studies will be necessary to clarify the functional impact of these variants on the gene product and DNA damage repair. According to the Polyphen-2 database (genetics.bwh.harvard.edu) five out of eight FA variants were defined probably or possibly damaging DNA repair (Supplementary Table 2). Functional studies using constructs including the mutated sequences may help to answer this question.^13^

Our data may indicate that heterozygous carriers of FA variants may have increased susceptibility to environmental carcinogens and to the DNA-damaging action of cytotoxic therapy used to treat primary tumors, leading to *de novo* or secondary leukemogenesis. In this line, a trend towards higher DEB-induced chromosome breakage and an increased release of fragmented DNA after exposure to X-irradiation have been reported in FA heterozygotes relative to controls.^14^

We have previously reported that t-MN secondary to lymphoproliferative diseases were characterized by a very low frequency or absence of epigenetic and spliceosome gene mutations, compared to t-MN following solid tumors.^15^ As the frequency of FA germline variants in this study did not appear to be different between lymphoproliferative neoplasms and breast cancer and between *de novo* and t-MN, the probable functional role of these variants in carcinogenesis does not appear to be restricted to a specific type of primary cancer or its treatment.

In the near future, we might have to revise our concept of genetic susceptibility to AML, taking into account not only recurrent genetic variants, such as polymorphisms, but also rare individual germline variants in critical genes.

## Figures and Tables

**Table 1:** Patient characteristics

*Patient characteristics*	n *(range)*
Median age (years, median, range)	63 (30–78)
Sex (M/F)	9/28
	
*Type of t-MN according to the WHO*	
* *AML	18
* *MDS	19
* *RA	6
* *RAEB-1	4
* *RAEB-2	9
	
*Primary malignancy*	
* *Hodgkin lymphoma	7
* *Non-Hodgkin lymphoma	12
* *Breast cancer	13
* *Breast and another cancer	5
	
*Karyotype (n=32 pts)*	
* *Normal	8
* *Complex	11
* *Isolated Chr. 7 abnorm.	4
* *Chr. 5+Chr. 7 abnorm.	3
* *11q23	1
* *Balanced translocation	2
* *Other	3
	
*Treatment for primary malignancy*	
* *CHT	23
* *RT	1
* *RT+CHT	13
Median latency between primary cytotoxic therapy and t-MN diagnosis (years)	6 (1.3–32.5)
Median overall survival (months)	8 (0.1–88)

Abbreviations: AML, acute myeloid leukemias; CHT, chemotherapy; MDS, myelodysplastic syndromes; RA, refractory anemia; RT, radiotherapy.

**Table 2:** Characteristics of t-MN patients carriers of FANC variants

*UPN*	*Primary tumor, age*	*t-MN latency, karyotype*	*Gene symbol and AA change*	*NCBI rs and/or COSMIC numbers*	*Control tissue*
*6531*	*Breast, 57 years*	*72 months, del5q31*	*FANCA L6F*	*rs189841793*	*Breast cancer and normal breast tissue*
*3155*	*NHL, 69 years*	*66 months, complex*	*FANCA S90T*	*Novel*	*NHL biopsy*
*5993*	*HL, thyroid, 52 years*	*48 months, Del7*	*FANCD2 T1376A*	*Novel*	*HL biopsy and normal lymphnode*
*6190*	*HL, 47 years*	*208 months, Trisomy 8*	*FANCD2 P256S*	*Novel*	*Buccal swab and hair follicle*
*6828*	*HL, 60 years*	*112 months, n.a.*	*FANCD2 M1023V;*	*Novel*	*HL biopsy*
*2885*	*Breast/ adrenal, na*	*na*	*FANCJ I364V FANCC L36F*	*Novel*	*Not available*

Abbreviations: HL, Hodgkin lymphoma; NHL, Non-Hodgkin lymphoma; t-MN, therapy-related myeloid neoplasms. NCBI and Cosmic database variant number and the reported frequency is indicated.
