# Supplementary material for: Fanconi anemia gene variants in therapy-related myeloid neoplasms
Source: Blood Cancer J. 2015 Jul 3;5(7):e323–. doi: 10.1038/bcj.2015.44 (PMC4526773; doi:10.1038/bcj.2015.44)
Supplement: Supplementary Information [file bcj201544x1.doc]

**Supplementary Table 1 .** Primer sequences and annealing temperature used for validations of mutations, using Pyrosequencing and Sanger sequencing.

| **Gene** | **Primers sequences** | **Annealing temperature** | **Technique** |
| --- | --- | --- | --- |
| **FANCA L6F** | Fwd: BIO-CCC CGT GTG TGA ATT GTG C  Rew: TTG ACC CTT CCC GCT ACG  Seq: CCG CTA CGG AGA GAA | 62°C | Pyrosequencing |
| **FANCA S90T** | Fwd: GTT TGT TGT GTT TTG GAT TCT AGG  Rew: BIO-ACC CTT CTG CAA TTC AAA ATA GAG  Seq: GAG GCC TAT GCT AAT CA | 62°C | Pyrosequencing |
| **FANCA S90T P497L** | Fwd: TTG TGT TTT AAT TTT ACT AAT GG  Rew: AAC TCA AGA GTC AAA AGA AA | 62°C | Sanger |
| **FANCC L36F** | Fwd: BIO-AAG CTT TCT GTA TGG GAT CAG G  Rew: TAC CAT CTC TTT CAA GGC TTC ATA  Seq: ACT GAG CCA CGT GAA | 62°C | Pyrosequencing |
| **FANCD2 M1023V** | Fwd: GAG ATC TGC CCA AGA AAT TGT TCA  Rew: BIO- TGA ATG TTC TCC AGG TGG TTA CAC  Seq: CAA CTG CTG ACC CCA | 62°c | Pyrosequencing |
| **FANCD2 P256S** | Fwd: TCC CAA TCC TGG ATG TCC T  Rew: BIO-AGG CAA TGA CTG ACT GAC ACT TGT  Seq: AGC CTC CGA CTT GAC | 60°C | Pyrosequencing |
| **FANCD2 T1376A** | Fwd: ACG AGA CTC ACC CAA CAT GTG C  Rew: BIO- TCC CGG TTT TTT AGA TTG CCC  Seq: CAG AGT CAA AGC TAT GC | 62°C | Pyrosequencing |
| **FANCJ I364V** | Fwd: CAC AGC CCG AGA ACT AAT ACA AGA  Rew: BIO-TTT CCC TTA TTT GTG CAT CTA GAA  Seq: GAA CTA ATA CAA GAT GCT GA | 62°C | Pyrosequencing |
| **FANCJ P47L** | Fwd: AGT TCC TTC TGC TAT TGT TGT GTT  Rew: CGA TCC TAC CAC CTC AGT CT | 62°C | Sanger |
| **FANCL T372 fs** | Fwd: GTG AAT GTC CAT ATT GTA GTA AGG TAA GC  Rew: CCA GTC CAG ATA TAT TCA AGA AGT CAA GA | 62°C | Sanger |

**Legend.**

**BIO:** biotinylated oligo 5 '; **fs:** frameshift.

| **Gene** | **Mutation** | **Region name (NCBI)** | **Function (NCBI)** | **Putative effect (PolyPhen-2)** | **Score**  **(0-1)** |
| --- | --- | --- | --- | --- | --- |
| FANCJ | I364V | RAD 3 | Conserved Domain | Probably damaging | 0,997 |
| FANCD2 | P256S |  | Interaction with FANCE and BRCA2 domain | Benign | 4 |
| FANCD2 | M1023V | FANC | Fanconi Anemia ID complex proteins FANCI and FANCD2 | Benign | 0.162 |
| FANCD2 | T1376A | FANC | Fanconi Anemia ID complex proteins FANCI and FANCD2 | Benign | 1 |
| FANCC | L36F | FANCONI C | Conserved Domain | Possibly damaging | 0.638 |
| FANCA | L6F | Not reported | Not reported | N/A | N/A |
| FANCA | S90T |  |  | Probably damaging | 0.953 |
| FANCA | P497L |  |  | Probably damaging | 1.000 |
| FANCL | T372 fs |  |  | N/A | N/A |
| FANCJ | P47L |  | Helicase motif | Probably damaging | 1.000 |

**Supplementary Table 2.** Putative functions of FA gene mutations, according to the PolyPhen-2 database. N/A: Not available.

**fs:** frameshift.
